# Mitogen-Activated Protein Kinase *CaDIMK1* Functions as a Positive Regulator of Drought Stress Response and Abscisic Acid Signaling in *Capsicum annuum*

**DOI:** 10.3389/fpls.2021.646707

**Published:** 2021-04-29

**Authors:** Minchae Kim, Soongon Jeong, Chae Woo Lim, Sung Chul Lee

**Affiliations:** Department of Life Science (BK21 program), Chung-Ang University, Seoul, South Korea

**Keywords:** ABA, drought, kinase, phosphorylation, stomata

## Abstract

Protein phosphorylation by kinase is an important mechanism for adapting to drought stress conditions. Here, we isolated the *CaDIMK1* (*Capsicum annuum drought-induced MAP kinase 1*) from dehydrated pepper leaf tissue and functionally characterized it. Subcellular localization analysis revealed that the CaDIMK1 protein was localized in the cytoplasm and nucleus. *CaDIMK1*-silenced pepper plants exhibited drought-susceptible phenotypes that were characterized by increased transpiration rates, low leaf temperatures, and decreased stomatal closure. In contrast, *CaDIMK1*-overexpressing (OX) transgenic *Arabidopsis* plants were hypersensitive to abscisic acid (ABA) from germination to adult growth stages. Furthermore, the *CaDIMK1*-OX plants were tolerant to drought stress. The transcript levels of several stress-related genes were high in *CaDIMK1*-OX plants than in wild-type plants. Taken together, our data demonstrate that *CaDIMK1* acts as a positive modulator of drought tolerance and ABA signal transduction in pepper plants.

## Introduction

Sessile plants are exposed to various environmental stresses that can lead to decreased crop yields. Water deficits caused by cold temperature, high salinity, and drought stresses can impact plant survival. Water is a key factor for plant growth, development, and survival. Plants have adapted to water-deficit conditions by altering many survival processes such as stomatal closure, stress-related gene expression, and abscisic acid (ABA) accumulation (Golldack et al., 2014; Basu et al., 2016; Ullah et al., 2018).

The phytohormone ABA plays a critical role in response to abiotic stress as well as plant growth and development (Finkelstein et al., 2002; Tuteja, 2007). When plants encounter a water-deficit condition, ABA is synthesized in several plant tissues, in particular in the leaves, leading to the initiation of signal transduction associated with a plant-adaptive response (Schroeder et al., 2001; Vahisalu et al., 2008; Lee et al., 2009, 2013). When the guard cells recognize ABA, the turgor and volume of the guard cells decrease, resulting in stomatal closure (Kim et al., 2010; Dong et al., 2018). The core ABA signal transduction pathway is composed of ABA receptors (PYR/PYL/RCAR) that directly bind to ABA and perceive ABA signals (Gonzalez-Guzman et al., 2012; Dittrich et al., 2019). This complex recognizes clade A protein phosphatase 2Cs (PP2Cs), including AHG1, PP2CA, HAB1, HAB2, ABI1, ABI2, AIP1, AIP2, and AIP3, and in turn inhibits phosphatase activity. The interactions between PP2Cs and PYR1/PYLs/RCARs lead to the release of the SnRK2 type kinases from PP2Cs, which activate downstream signaling, including transcription factors and ion channels (Brandt et al., 2012; Komatsu et al., 2013; Lim et al., 2015; Shinozawa et al., 2019). The biological functions of ABA have been widely studied; however, the precise mechanisms for ABA signaling and ABA-mediated drought stress remain largely unexplored.

All eukaryotes evolutionarily have the mitogen-activated protein kinase (MAPK) signaling module, which is associated with the regulation of plant growth, development, and stress response (Rodriguez et al., 2010; Xu and Zhang, 2015; Bigeard and Hirt, 2018). Three protein kinases commonly constitute the MAPK cascade: MAPK, MAPK kinase (MAP2K), and MAPK kinase (MAP3K), which are linked in a variety of ways to specific upstream activators and downstream substrates (Jonak et al., 2002; Mishra et al., 2006). Many MAPK cascades play a role in response to abiotic stress (Rodriguez et al., 2010), and its activation has been reported to be associated with ABA in various plant species (Danquah et al., 2014; de Zelicourt et al., 2016). Previous studies have revealed the components of MAPK cascades: 60 MAP3Ks, 20 MAPKs, and 10 MAP2Ks in *Arabidopsis* (Ichimura et al., 2002). The MAP3Ks constitute the largest group of kinases in the MAPK cascade and are classified into three groups: Raf-like kinase, ZR1-interacting kinase (ZIK), and MEKK (Jonak et al., 2002). In abiotic stress responses, several MAP3Ks are involved with the ABA core signaling pathway. Recently, MAP3Ks were found to be part of the activation of some SnRK2-type kinases through ABA-dependent and ABA-independent manner (Lin et al., 2020; Soma et al., 2020; Takahashi et al., 2020). Group A PP2C ABI1 interacts with MAP3K18 and inhibits its kinase activity (Mitula et al., 2015). Additionally, AIK1/MKKK20 modulates ABA sensitivity in terms of guard cell signaling, primary root growth, and development in *Arabidopsis* (Li Y. et al., 2017), and MAP3K YDA/YODA plays an essential role in stomatal patterning and inflorescence development (Bergmann et al., 2004; Wang et al., 2007). Loss-of-function mutants of *AIK1/MKKK20* exhibit an increased number of stomata, consistent with clustered stomata in loss-of-function mutant of YDA/YODA.

In this study, the pepper MAP3K/MEKK gene, *CaDIMK1* (*Capsicum annuum drought-induced MAP kinase 1*) was identified, which was highly induced by drought stress. ABA also increases the transcript level of *CaDIMK1*. *CaDIMK1*-silenced peppers and *CaDIMK1*-overexpressing (OX) transgenic *Arabidopsis* plants showed altered phenotypes to drought stress and ABA treatments, accompanied by different transpiration rates and stomatal apertures. *CaDIMK1*-OX plants also displayed an ABA hypersensitivity in germination and seedling growth stages. These data demonstrate that CaDIMK1 acts as a positive modulator in response to drought stress and ABA.

## Materials and Methods

### Plant Materials and Growing Conditions

In this study, *Arabidopsis thaliana* ecotype Columbia-0 was used for the OX transgenic plants. Seeds were disinfected with 70% ethanol and planted on MS plates with 0.5% sucrose. After cold stratification (4°C) for 2 days, seeds of each line were germinated at 24°C and 40% humidity for 7 days. The seedlings were then transplanted into plastic pots containing vermiculite, perlite, and peat moss (9:1:1 ratio). Pepper (*C. annuum* cv. Nockwang) and tobacco (*Nicotiana benthamiana*) plants were grown in pots containing a 1:1:1 ratio of a compost soil mix (vermiculite, perlite, and peat moss, 2:3:5, v/v/v), loam soil, and sand. All seedlings were grown under the following conditions: 24 ± 1°C, 60% humidity, and long-day condition (light/dark: 16 h/8 h).

### Generation of Overexpression Transgenic Plants in *Arabidopsis*

The *CaDIMK1* coding region was amplified using primer pairs (Supplementary Table 1). The PCR products of *CaDIMK1* were cloned to the entry vector (pENTR/D-TOPO; Invitrogen) for gateway cloning and then subcloned to the destination vector for making fusion protein with green fluorescent protein (GFP) through LR reaction. The *35S:CaDIMK1-GFP* plasmid was introduced to the strain GV3101 of *Agrobacterium tumefaciens* by electroporation. For plant transformation, we used the floral dip method (Clough and Bent, 1998). Homozygous T3 transgenic seeds were grown in selective media containing 50 μg ml^–1^ of phosphinothricin for further studies.

### Virus-Induced Gene Silencing in Pepper Plants

Virus-induced gene silencing (VIGS) assay was performed to generate *CaDIMK1*-silenced pepper plants using the tobacco rattle virus (TRV) as previously described (Liu et al., 2002). Briefly, a 411–710-bp region of *CaDIMK1* was amplified using the specific primers (Supplementary Table 1), which were ligated into a pTRV2 vector. The strain GV3101 of *A. tumefaciens* harboring constructs was infiltrated by syringe in both cotyledons of the pepper plant (each construct: OD600 = 0.2).

### Protein Localization Assay

For the protein localization assay, GFP-tagged *CaDIMK1* was transiently expressed in *N. benthamiana* through agroinfiltration. The GFP fluorescence signals were detected using LSM700 confocal microscope (Carl Zeiss) 2 days after infiltration.

### Abscisic Acid, Drought, NaCl, and H_2_O_2_ Treatments

We treated with either a 100-μM ABA or 100-μM H_2_O_2_ solution and irrigated with a 200-mM NaCl solution in 4-week-old pepper leaves to analyze the induction of *CaDIMK1* transcripts in pepper plants. Two-week-old pepper plants were treated with drought stress by withholding watering, and the dehydrated leaves were harvested at 0, 8, 10, and 12 days after treatment. For *Arabidopsis*, 3-week-old plants were applied with drought stress by removing them from the soil, followed by the leaves being harvested at the indicated time.

### Germination Test and Seedling Growth Assay

For a germination test, seeds of each genotype were sown on 1/2 MS agar plates with 0.5, 0.75, and 1.0 μM ABA. The germinated seeds (radicle emergence) were measured daily for 6 days. Five days after plating, the numbers of seedlings with fully expanded green cotyledons were counted, and the root lengths were measured.

### Stomata Aperture Assay and Thermal Imaging Analysis

To measure the stomatal aperture, we collected leaf peels from 4-week-old pepper (TRV2:*CaDIMK1* and TRV2:00) plants and 3-week-old *Arabidopsis* (*CaDIMK1*-OX line #1, line #2, and wild-type) plants cultivated under well-watered condition. The collected leaf peels were incubated on stomata open buffer (SOB) for 3 h under a light intensity of 100 μmol m^–2^ s^–1^ to open the stomata fully. After being transferred into a new SOB containing various concentrations of ABA to induce stomata closing, stomata were observed using a Nikon Eclipse 80i microscope. The ratio of stomatal aperture width to length was calculated from at least 100 stomata of each plant line using ImageJ.

Pepper and *Arabidopsis* plants at the same development stage were applied with 50 μM ABA for 4 h to analyze leaf temperature changes in response to ABA. Thermal images of each plant line were taken by a T420 thermal imaging camera (FLIR systems).

### Water Loss Measurement

The leaf tissues from 4-week-old pepper (TRV2:*CaDIMK1* and TRV2:00) plants and rosette leaves from 3-week-old *Arabidopsis* (*CaDIMK1*-OX line #1, line #2, and wild-type) were harvested and placed in a growth chamber. Transpirational water losses of pepper and *Arabidopsis* were examined by measuring the fresh weight of leaf samples during 10 and 7 h, respectively, after detachment.

### RNA Isolation and Semiquantitative Reverse Transcription-Polymerase Chain Reaction (RT-PCR) and Quantitative RT-PCR Assay

RT-PCR analysis was performed using plant total RNA samples isolated from 3-week-old pepper or 3-week-old *Arabidopsis* plants using TRI reagent (Invitrogen). For the removal of contamination of genomic DNA, RNA samples were applied with DNase. cDNA was synthesized by using iScript cDNA synthesis kit with 1 μg RNA and oligo-dT primers (Bio-Rad). *CaDIMK1* and its homologous genes were amplified using primer pairs (Supplementary Table 1).

The expression patterns of stress-induced genes were analyzed using quantitative RT-PCR (qRT-PCR) (CFX96 Touch^TM^RT PCR detection system; Bio-Rad) with the iQ^TM^ SYBR Green Supermix (Bio-Rad). qRT-PCR was conducted following the manufacturer’s instructions. *C. annuum* Actin1 (*CaACT1*) and *A. thaliana* Actin8 (*AtACT8*) were used as an internal control for the normalization.

### Protein Expression, Purification, and *in vitro* Kinase Assay

The expression and purification of GST-tagged *CaDIMK1*, *CaDIMK1*^K32N^, and OST1 recombinant proteins in bacterial cells were conducted as previously described (Lim et al., 2017). Briefly, the coding regions of each gene were inserted into a GST tagging *Escherichia coli* expression vector (pGEX4T-3), which were transferred into strain BL21 of *E. coli* cells. The GST-tagged proteins were expressed and purified by the glutathione S-transferase (GST) gene fusion system following the manufacturer’s instructions (GE Healthcare Bio-Sciences).

An *in vitro* kinase analysis used recombinant proteins that were reacted in a phosphorylation buffer (1 mM CaCl_2_, 1 mM dithiothreitol, 2.5 mM MgCl_2_, 2.5 mM MnCl_2_, and 20 mM Tris–HCl) with [γ-^32^P] ATP (7.5 μCi). After incubation at 30°C for 2 h, the reaction was terminated by boiling in a 5 × SDS-sample buffer with 25% β-mercaptoethanol, bromophenol blue (0.005%, G-250), glycerol (50%), SDS (10%), and Tris–HCl (250 mM, pH 6.8). The reacted kinases were separated using SDS-PAGE (10%). The SDS-PAGE gel was dried, and the phosphorylation signal was observed *via* autoradiography by Personal Molecular Imager (Bio-Rad).

## Results

### Isolation of Drought-Induced *CaDIMK1*

**FIGURE 1:**
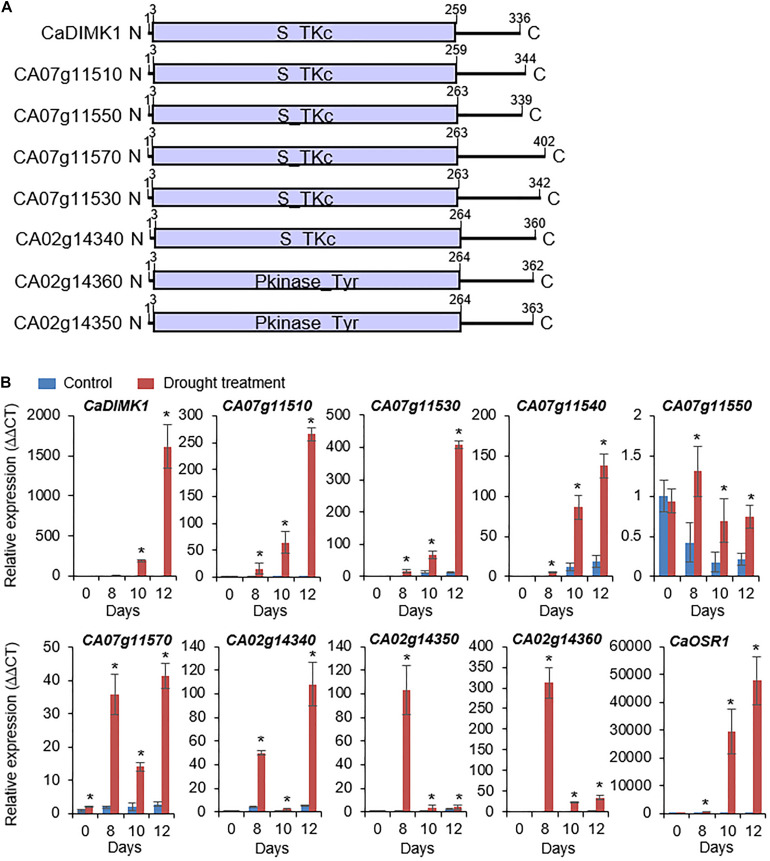
Drought-induced pepper MAP3 kinase genes. **(A)** Protein domain analysis of pepper *MAP3Ks* according to deduced amino acids. The kinase domain was marked *via* released data (web address: http://smart.embl-heidelberg.de). **(B)** Induction levels of *MAP3K* genes in pepper leaf tissue after drought stress treatment. The expression values are normalized by pepper *Actin1* (*CaACT1*) gene as an internal control, and the induction level of each gene at 0 day was 1.0. As a positive control for drought treatment, *CaOSR1* gene was amplified in parallel. Values are mean ± standard deviation, *n* = 3; asterisks indicate significant differences compared with nontreated control (Student’s *t*-test; *P < 0.05).

To isolate drought-induced MAP3 kinase, we used RNA-seq analysis and isolated eight MAP3 kinase genes from pepper plant leaves under drought stress: *CA07g11510*, *CA07g11520*, *CA07g11530*, *CA07g11540*, *CA07g11550* (Jeong et al., 2020), *CA07g11570*, *CA02g14340*, *CA02g14350*, and *CA02g14360* (Figure 1A). Domain analyses using a web-based tool (SMART)^1^ showed that MAP3Ks have a kinase domain, which phosphorylates tyrosine or serine-threonine amino acid residues of a target protein. We conducted qRT-PCR analysis to analyze the expression patterns of the MAP3Ks in the leaf tissues from pepper plants treated with drought stress. As shown in Figure 1B, all genes, except *CA07g11550*, were significantly induced by drought stress. From these genes, we selected *CA07g11520*, which had the highest differential expression level after drought treatment, for further investigation and was designated *CaDIMK1*.

**FIGURE 2:**
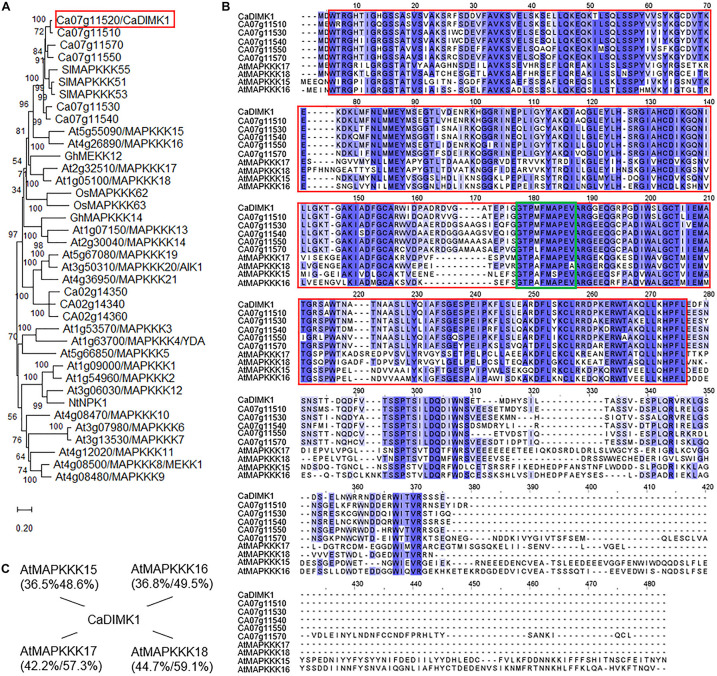
Amino acid sequence analysis of *CaDIMK1*. **(A)** Phylogenetic tree of pepper MAP3 kinases. The phylogenetic analysis was conducted *via* MEGA software (version 10.1) using the deduced amino acid sequences of MAP3 kinases from *Arabidopsis*, pepper, rice, cotton, tobacco, and tomato plants. The phylogenetic tree was built according to the neighbor-joining method, and bootstrap values were calculated from 1,000 bootstrap replications and are at each branch point. Scale bar indicates the evolutionary distance computed using the Poisson correction method. **(B)** Multiple alignment analysis of CaDIMK1 with its homologous *Arabidopsis* MAP3 kinases. Identical and similar amino acid residues are shaded according to the percentage identity in ClustalW2. Gaps introduced to maximize the alignment of homologous regions are marked by dashes. A red box indicates a serine/threonine protein kinase domain and a green box for a conserved kinase domain G(T/S)Px(W/Y/F)MAPEV in the MEKK-like group of the MAPKKK family. **(C)** Sequence homology of CaDIMK1 with its homologous *Arabidopsis* MAP3 kinases. In parenthesis, protein identity and similarity were calculated using EMBOSS needle (https://www.ebi.ac.uk/Tools/psa/emboss_needle/).

*Capsicum annuum drought-induced MAP kinase 1* is composed of 336 amino acids (1,011-bp open reading frame) with an isoelectric point of 5.74 and a molecular weight of 37.49 kDa. When performing the BLASTP search on NCBI^2^, we found that CaDIMK1 (accession no. KAF3666587.1) shares relatively high identities/similarities (46.74–81.85%/63.73–86.61%) with the MAP3K proteins, in particular belonging to MEKK subfamily, from several higher plant species. A phylogenetic tree analysis was conducted using MAPKKK-MEKK proteins of the model plant *Arabidopsis*, together with drought- and/or ABA-responsive MEKK kinases from rice, cotton, tobacco, and tomato, characterized in previous studies (Ichimura et al., 2002; Shou et al., 2004; Rao et al., 2010; Wu et al., 2014; Mao et al., 2019; Na et al., 2019; Zhang et al., 2020). As shown in Figure 2, CaDIMK1, CA07g11520, CA07g11530, CA07g11540, CA07g11550, and CA07g11570 were clustered with AtMAPKKK15/16/17/18, SlMAPKKK51/53/55, OsMAPKKK62/63, and GhMEKK12. In contrast, CA02g14340, CA02g14350, and CA02g14360 were sorted into the same clade with AtMAPKKK19, 20, and 21. Previous studies have revealed an ABA-induced regulation of those *Arabidopsis MAPKKK* gene expression (Wang et al., 2011; Danquah et al., 2015) and functional involvement of some genes in ABA signaling (Mitula et al., 2015; Li Y. et al., 2017). Also, AtMAPKKK18 and GhMEKK12 play a positive role in drought tolerance of *Arabidopsis* and cotton, respectively (Li K. et al., 2017; Zhang et al., 2020). Based on these results, we proposed that CaDIMK1 could be involved in plant responses to ABA and drought stress.

### Molecular Characterization of CaDIMK1

**FIGURE 3:**
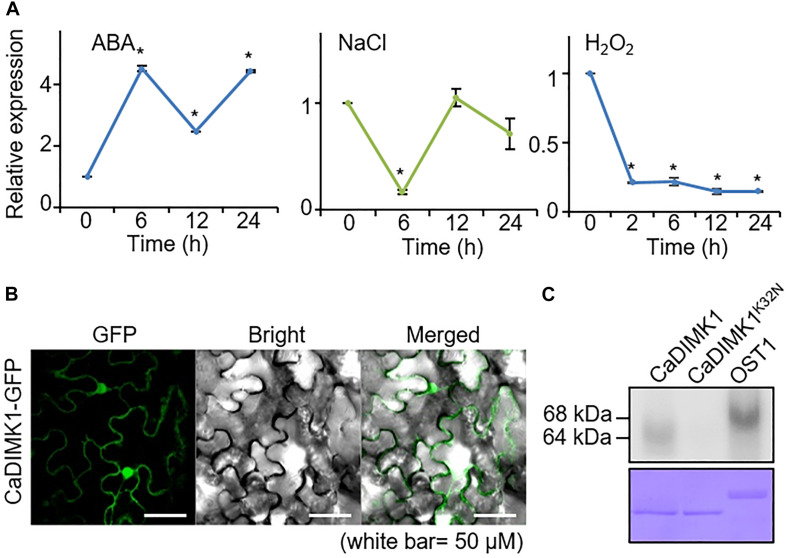
Expression of *CaDIMK1* gene and subcellular localization of CaDIMK1 protein. **(A)** Induction levels of *CaDIMK1* in pepper plant leaf tissue treated with abscisic acid (ABA; 100 μM), sodium chloride (NaCl; 200 mM), and hydrogen peroxide (H_2_O_2_; 100 μM). The expression values are normalized to pepper *Actin1* (*CaACT1*) gene as a standard control. The induction level of *CaDIMK1* at 0 h after treatment was 1.0. Values are mean ± SE, *n* = 3; asterisks indicate statistical differences compared with 0 h after treatment according to Student’s *t*-test (*P < 0.05). **(B)** Subcellular localization of *CaDIMK1* protein in the epidermal cells of *Nicotiana benthamiana*. The transient expression of 35S:*CaDIMK1-GFP* construct was expressed in *N. benthamiana* leaves and detected using a confocal microscope. **(C)** *In vitro* auto kinase assay of GST-*CaDIMK1* and GST-*CaDIMK1*^K36N^. [γ−−^32^P]-ATP was used for kinase assay. As a positive control, *Arabidopsis* OST1 was used. CBB, Coomassie brilliant blue staining.

We initially performed qRT-PCR analyses on pepper plants treated with ABA, NaCl, and H_2_O_2_ to determine whether *CaDIMK1* is associated with abiotic stress responses (Figure 3A). Following exposure to ABA, high induction of *CaDIMK1* expression started at 6 h. In contrast, NaCl treatment did not significantly affect the expression level of *CaDIMK1* at all time points, except at 6 h. *CaDIMK1* expression level showed a nine-fold decrease 2 h after treatment with H_2_O_2_. These results suggested that *CaDIMK1* is presumably involved in both ABA signaling and drought stress response.

Plant kinases are active in various areas within a cell; hence, we examined the localization of CaDIMK1 protein in the cell. When the fusion protein of *CaDIMK1* with GFP was expressed in the epidermal cells of tobacco leaf tissues, the detected GFP fluorescence suggests that *CaDIMK1* can function in the cytoplasm and nucleus (Figure 3B). CaDIMK1 has a serine-threonine kinase domain (Figure 1A); hence, we investigated the kinase activity of *CaDIMK1* through *in vitro* kinase analysis (Figure 3C). We used *CaDIMK1*^K32N^ with a substitution of lysine 32 for asparagine in the ATP-binding domain (Carrera et al., 1993) as a negative control and *Arabidopsis* OST1/SnRK2.6 as a positive control. As expected, auto kinase activity was shown in *CaDIMK1*, but not in *CaDIMK1*^K32N^.

### Hypersensitivity to Drought Stress in *CaDIMK1*-Silenced Pepper Plants

**FIGURE 4:**
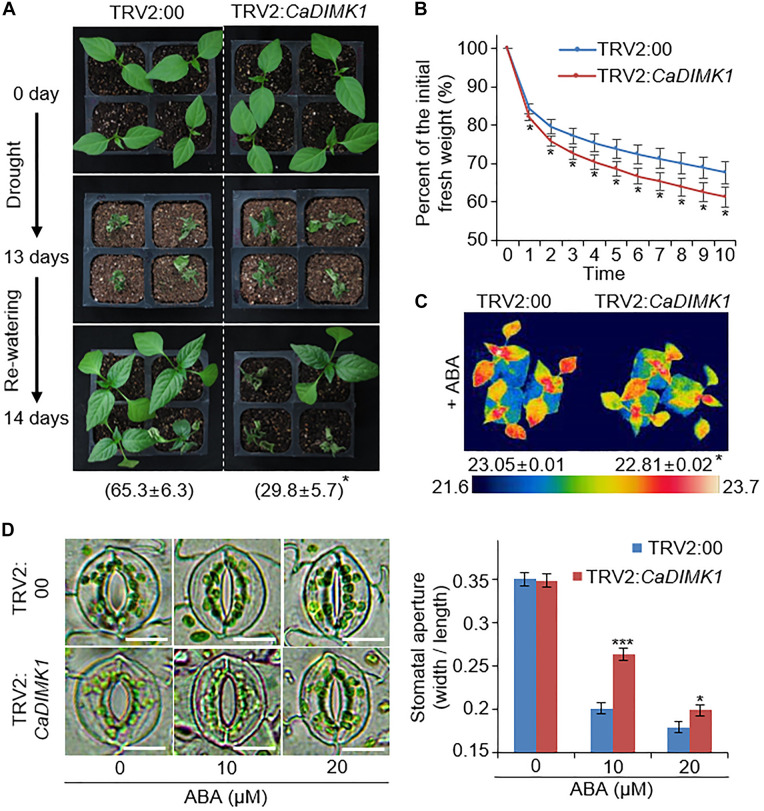
*CaDIMK1* mediated drought tolerance *via CaDIMK1*-silenced pepper plants. **(A)** Drought sensitivity of TRV2:*CaDIMK1* pepper plants. Three-week-old pepper plants expressed TRV2:00 and TRV2:*CaDIMK1* constructs were grown in well-watered conditions (upper). The pepper was exposed to drought stress by withholding watering for 13 days (middle), followed by 1 day after rewatering (lower). The survival rates were counted at 1 day after rewatering. **(B)** Reduced fresh weight from the detached leaf tissues of TRV2:00 and TRV2:*CaDIMK1* pepper plants during 10 h. **(C)** Reduced plant surface temperature of TRV2:*CaDIMK1* pepper plants after 50 μM ABA treatment. Values are mean ± SE with three independent experiments (*n* = 10). **(D)** Reduced stomatal apertures of TRV2:00 and TRV2:*CaDIMK1* pepper plants after various concentrations of ABA treatment. The stomata with guard cells were taken using a microscope when the stomatal pore size was measured. Leaves were incubated in stomata opening buffers with 0, 10, or 20 μM ABA. Values are mean ± SE with three independent experiments (*n* = 15). Asterisks indicate statistical differences between the TRV2:00 and TRV2:*CaDIMK1* pepper plants according to Student’s *t*-test (*P < 0.05, ***P < 0.001). The scale bar represents 10 μm.

*Capsicum annuum drought-induced MAP kinase 1* transcripts were considerably accumulated in the pepper leaves treated with drought stress and ABA (Figures 1B, 3A). Hence, we checked the functional role of *CaDIMK1 in vivo*. Since the pepper transformation has a technical limitation, we alternatively used the VIGS method in pepper plants and generated OX transgenic plants in *Arabidopsis* for the genetic studies of *CaDIMK1*. First, we produced *CaDIMK1*-silenced pepper plants (TRV2:*CaDIMK1*), showing a lower accumulation of *CaDIMK1* transcripts than control pepper TRV2:00 (Supplementary Figure 1A). To analyze how silencing of *CaDIMK1* affects pepper drought stress response, we subjected 2-week-old pepper plants of TRV2:00 and TRV2:*CaDIMK1* to drought stress by withholding watering for 13 days (Figure 4A). Plants grown under well-watered conditions did not show any different phenotypes (Figure 4A, upper panel). However, relative to the control pepper plants, the *CaDIMK1*-silenced pepper plants showed wilted phenotypes under drought stress. After recovery by rewatering (as indicated by 14 days), the survival rate of TRV2:*CaDIMK1* pepper plants (29.8 ± 5.7%) was dramatically lower than that of TRV2:00 pepper plants (65.3 ± 6.3%).

Preserving water by restricting transpirational water loss *via* stomata closure is critical for determining drought sensitivity. We measured the fresh weight of detached rosette leaves to investigate the rate of transpirational water loss. *CaDIMK1*-silenced pepper plants showed more significant water loss compared with the control plants (Figure 4B). In general, ABA treatment results in stomatal closing, causing enhanced leaf surface temperatures because of reduced evaporative cooling (Joo et al., 2019). We could not detect any differences in leaf surface temperatures between TRV2:*CaDIMK1* and TRV2:00 plants under well-watered conditions. However, leaf surface temperatures in TRV2:*CaDIMK1* were low compared with those in TRV2:00 plants following ABA treatment (Figure 4C). Consistently, there were no differences in the stomatal aperture between the TRV2:*CaDIMK1* and TRV2:00 plants without ABA (Figure 4D). The application of ABA induced stomatal closing in both plants; however, the pore sizes in TRV2:*CaDIMK1* plants were much larger than those in TRV2:00 plants. These results suggest that the downregulation of *CaDIMK1* conferred reduced drought resistance *via* modulating the rate of water loss and ABA-mediated stomatal closing.

### Increased ABA Sensitivity in *CaDIMK1*-OX Plants

**FIGURE 5:**
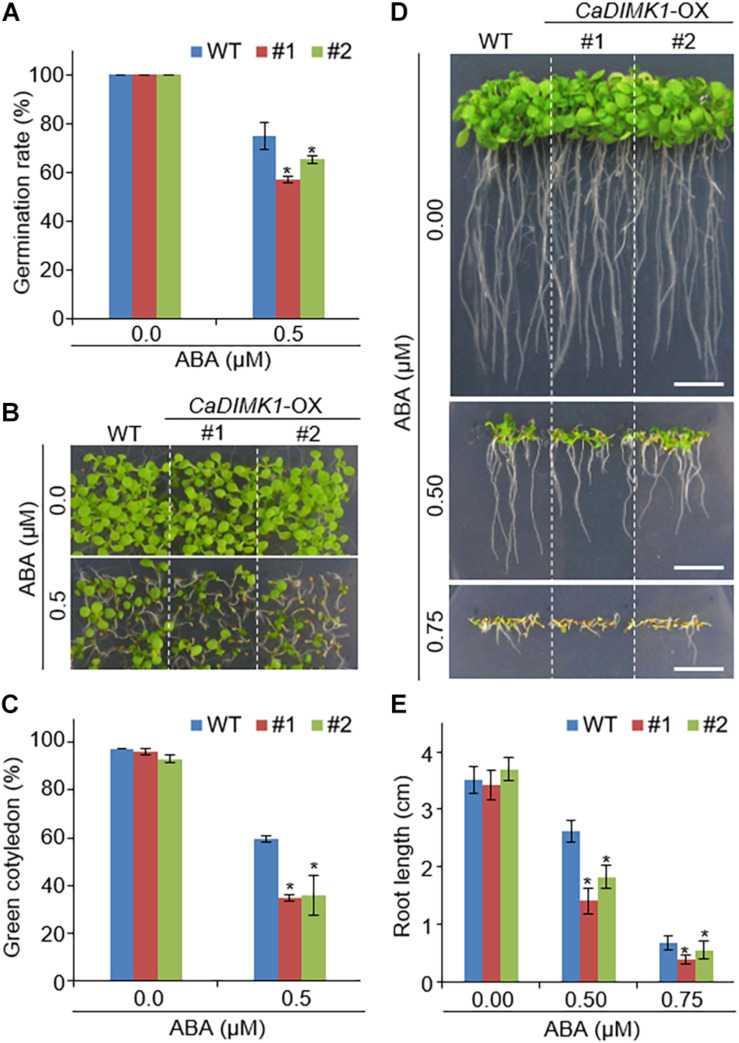
Increased sensitivity of *CaDIMK1*-OX plants to ABA. **(A)** Seed germination rates of transgenic lines and wild-type (WT) Col-0 plants on 0.5 × MS medium without ABA or supplemented with ABA and measured 7 days after sowing. **(B,C)** Cotyledon greening of *CaDIMK1-*OX and wild-type plants on 0.5× MS medium with or without ABA. Five days after plating, representative photographs were taken **(B)** and cotyledon greening in transgenic and wild-type plants was measured **(C)**. Values are mean ± SE with six independent experiments (*n* = 36). **(D,E)** Root length of *CaDIMK1-*OX *Arabidopsis* and wild-type (WT) plants on 0.5 × MS medium containing 0.0, 0.5, and 0.75 μM ABA. Seven days after plating, representative photographs were taken **(D)** and primary root lengths in the transgenic and wild-type plants were measured **(E)**. The scale bar represents 1 cm. Values are mean ± SE with three independent experiments (*n* = 25). Asterisks indicate statistical differences between wild-type and *CaDIMK1-*OX plants according to Student’s *t*-test (*P < 0.05).

**FIGURE 6:**
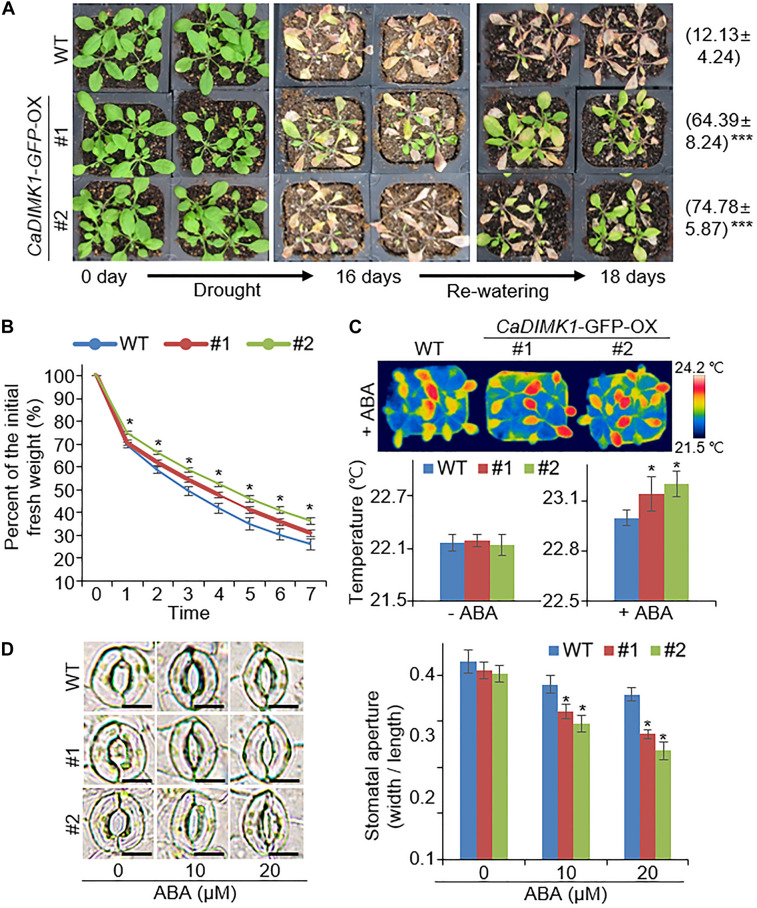
Increased drought stress tolerance of *CaDIMK1*-overexpressing (OX) plants. **(A)** Drought-tolerant phenotype of *CaDIMK1*-OX plants. Wild-type (WT) and *CaDIMK1*-OX *Arabidopsis* plants were cultivated for 3 weeks under well-watered conditions (left). *Arabidopsis* was exposed to drought stress by withholding watering for 16 days (middle), followed by 2 days after rewatering (right). The survival rates were counted 2 days after rewatering. Values are mean ± SE with three independent experiments (*n* = 42). **(B)** Transpiration water loss from wild-type and *CaDIMK1*-OX plant leaves during 7 h after leaf detachment. **(C)** Increased leaf surface temperatures of wild-type and *CaDIMK1*-OX *Arabidopsis* plants treated with 50μM ABA at 0 and 4 h. Values are mean ± SE with three independent experiments (*n* = 10). **(D)** Reduced stomatal opening in wild-type and *CaDIMK1*-OX transgenic plants treated with ABA. The stomata with guard cells were taken using a microscope when the stomatal pore size was measured. Leaves were incubated in stomata opening solutions with 0, 10, or 20 μM ABA. Values are mean ± SE with three independent experiments (*n* = 20). Asterisks indicate statistical differences between the wild-type and the transgenic plants according to Student’s *t*-test (*P < 0.05). The scale bar represents 10 μm.

We generated *CaDIMK1-* OX (*CaDIMK1-*OX) *Arabidopsis* transgenic plants to analyze the function of *CaDIMK1* in response to drought stress and ABA (Supplementary Figure 1B). Two independent lines (#1 and #2) were selected for further genetic assays. Compared with wild-type *Arabidopsis* plants, we did not detect any statistical difference in phenotypes at all growth stages (Figures 5, 6). First, we investigated seed germination and seedling growth of *CaDIMK1-*OX plants in response to ABA. *CaDIMK1-*OX and wild-type seeds were normally germinated in the 0.5 × MS media in the absence of ABA. Application of ABA inhibited the seed germination of both plant lines, but the *CaDIMK1*-OX line had a lower germination rate than the wild-type plant at 7 days after plating (Figure 5A). The number of expanded cotyledons were significantly higher in *CaDIMK1-*OX lines than in the wild-type plants (Figures 5B,C). When seedlings of the two plant lines were vertically grown, the primary root growths of *CaDIMK1-*OX seedlings were significantly longer than those of wild-type plants (Figures 5D,E). These data indicated that enhanced expression of *CaDIMK1* led to increased sensitivity to ABA in *Arabidopsis* seed germination and seedling stages.

### Increased Drought Resistance in *CaDIMK1*-OX Plants

**FIGURE 7:**
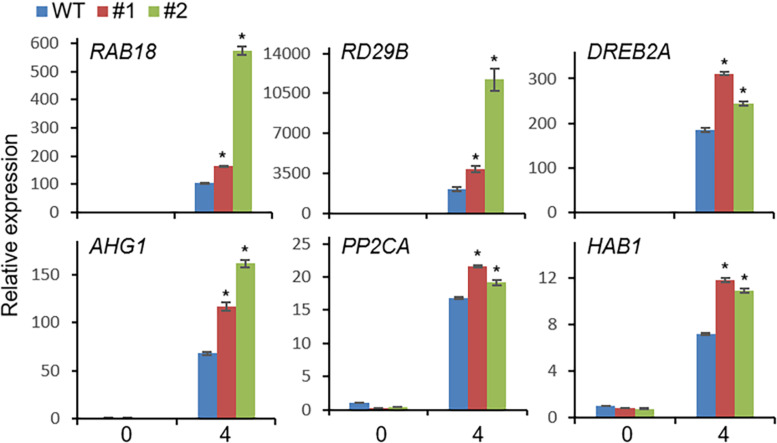
qRT-PCR assay of stress-responsive genes in *CaDIMK1*-overexpressing (OX) plants treated with drought stress at 0 and 4 h. The expression values are normalized to the *Actin8* genes as an internal control. Values are mean ± SE, n = 3. Asterisks indicate statistical differences between wild-type and transgenic plants according to Student’s *t*-test (*P < 0.05).

Based on the drought-induced expression of *CaDIMK1* and the reduced drought resistance of *CaDIMK1*-silenced pepper (Figures 1, 4), we tested how overexpression of *CaDIMK1* affects drought resistance in *Arabidopsis* plants (Figure 6). There were no differences between *CaDIMK1*-OX and wild-type plants under well-watered conditions (Figure 6A). Drought stress was applied by withholding watering for 16 days. Compared with wild-type plants, *CaDIMK1-*OX plants withered less, and more *CaDIMK1*-OX plants survived at 2 days after rewatering. The survival rate of *CaDIMK1*-OX was 64.39–74.78%, whereas that of wild-type plants was only 12.13% (Figure 6A). When measuring the water loss rate in rosette leaf tissues during 0–7 h after leaf detachment, we found that the fresh weight loss of *CaDIMK1-*OX leaves was significantly lower than that in the wild-type leaves (Figure 6B). To determine if this enhanced drought resistance is associated with ABA-mediated regulation of stomatal closure, we analyzed changes in leaf surface temperatures and stomatal apertures in response to ABA. Both *CaDIMK1*-OX and wild-type plants showed similar leaf temperatures under normal growth conditions; however, after ABA treatment, the leaf surface temperatures of the *CaDIMK1-*OX mutants were higher than those of the wild-type plants (Figure 6C). Consistently, the stomatal pore sizes of *CaDIMK1-*OX plants were smaller than those of wild-type plants after ABA treatment (Figure 6D). We also measured the transcript level of stress-responsive genes such as *RAB18*, *RD29B*, *DREB2A*, *AHG1*, *PP2CA*, and *HAB1*. qRT-PCR analyses revealed that these stress-responsive genes were highly induced in *CaDIMK1*-OX leaves than in wild-type leaves after treatment with drought stress (Figure 7). As shown by phenotypic analysis, *CaDIMK1* plays an essential role in drought resistance by controlling ABA-dependent stomatal apertures and ABA-responsive gene expression.

## Discussion

Plants modulate cellular activities using several processes such as transcription and posttranslational modifications to survive drought stress. Phosphorylation mediated by protein kinase is one of the posttranslational modifications, which plays a critical function in abiotic stress response and ABA signaling (Shi et al., 2018; Wang et al., 2018; Hong et al., 2020; Jeong et al., 2020). Previous studies reported that many protein kinases related to stress response were identified and functionally characterized; however, the exact process and function of these proteins remain elusive. In the ABA signal transduction pathway, SnRK2-type kinases are core components and act as positive regulators of drought stress response and ABA signal transduction pathway *via* modulation of stress-responsive gene transcription and channel activity (Geiger et al., 2009; Joshi-Saha et al., 2011; Brandt et al., 2012). Receptor-like kinases (RLKs) act as positive and negative regulators in the drought stress response (Ouyang et al., 2010; Hua et al., 2012; Marshall et al., 2012). RLKs perceive signals from intercellular spaces and transfer amplified signals to downstream substrate proteins (Shiu and Bleecker, 2003; Gish and Clark, 2011; Liang and Zhou, 2018). MAP kinase is also associated with plant responses to biotic and abiotic stresses (de Zelicourt et al., 2016; Zhang et al., 2016; Zhao et al., 2017); however, the exact functions in stress responses have been less studied than SnRK2-type kinases and RLKs. MAPK cascades are intracellular signaling pathways with sequential phosphorylation reactions to activate downstream partners in response to external signals (Rodriguez et al., 2010; Xu and Zhang, 2015). As revealed by physiological and molecular analysis, we have identified in this work the drought-induced pepper MEKK gene *CaDIMK1*, which plays an essential function in ABA signal transduction and drought response.

Plants initiate defense mechanisms under water-deficit conditions, such as accumulation of ABA and stress-responsive genes (Sato et al., 2018; Lu et al., 2019). Owing to low transformation efficiency in pepper plans, the VIGS analysis in pepper plants and overexpression assay in *Arabidopsi*s were used for genetic investigation in this study. ABA accumulation in leaf tissue restricts transpirational water loss by closing the stomata, conferring drought tolerance. Downregulated *CaDIMK1* by VIGS in pepper plants displayed a hypersensitive phenotype to drought stress, accompanied by large stomatal apertures that increase evaporation rates (Figure 4). Conversely, *CaDIMK1*-OX *Arabidopsis* displayed a drought-tolerant phenotype, which reduced transpirational water loss and stomatal pore size (Figure 6). These phenotype analyses suggest that different stomatal pore sizes in the silenced and overexpressed plants modulate water consumption, leading to altered drought phenotypes. Together with ABA signaling and drought stress, CaDIMK1 could be involved in different stress responses, including high salinity and osmotic stress, based on the altered expression of *CaDIMK1* by treatment with NaCl and H_2_O_2_ (Figure 3A). Both drought stress and high salinity decrease the water availability to plant cells and also cause the accumulation of ROS such as hydrogen peroxide (Hasegawa et al., 2000). As homologs of CaDIMK1, *AtMAPKKK15/16/17/18* are salt-inducible MEKK genes (Choi et al., 2017). In rice, *OsMAPKKK63* is also induced by salt and its loss-of-function mutant shows decreased tolerance to salt stress (Na et al., 2019). Although *CaDIMK1* gene expression in pepper leaves transiently decreased by salt stress compared with those genes, its functional involvement in response to salt stress may be supported by the data showing that *CaDIMK1*-OX plants were less sensitive to salt and mannitol during germination and seedling growth (Supplementary Figure 2).

The expression levels of stress- or ABA signal transduction-related genes are necessary to overcome drought stress, leading to plant survival (Zhu, 2016; Sato et al., 2018; Sharma et al., 2018; Lu et al., 2019; Joo et al., 2020). In this present study, the downstream substrate proteins of *CaDIMK1* were not found; however, relative to wild-type plants, the transcript levels of stress- or ABA signal transduction-related genes were higher in *CaDIMK1*-OX plants. This indicates that *CaDIMK1* may regulate and act upstream of these genes. In the ABA signaling pathway, clade A PP2Cs are core components that negatively control ABA signal transduction through dephosphorylation of SnRK2-type kinases (Robert et al., 2006; Nishimura et al., 2007; Umezawa et al., 2009; Vlad et al., 2009; Antoni et al., 2012). *Arabidopsis* clade A PP2C ABI1 also inhibits MAP3K protein MAPKKK18 and affects the stability of this kinase (Mitula et al., 2015). Interestingly, *CaDIMK1*-OX plants showed upregulation of clade A PP2Cs, including *AHG1*, *PP2CA*, and *HAB1*. Based on the relationship between MAP3Ks and clade A PP2Cs at the transcriptional and posttranslational levels, we proposed that CaDIMK1 may function upstream of clade A PP2Cs or CaDIMK1-mediated induction of *PP2C* genes may be part of the negative feedback regulation of the ABA signaling pathway. Under normal growth conditions, the phenotypes of the *CaDIMK1*-OX *Arabidopsis* plants and the expression levels of stress-related genes were not indistinguishable. Hence, the identification of processes that are upstream and downstream of *CaDIMK1* will help comprehend the *in vivo* role of *CaDIMK1* in plant cell to overcome drought stress.

In summary, altering the expression of *CaDIMK1* affected seed germination, seedling growth, and drought stress response. This study suggests that *CaDIMK1* is a positive regulator of ABA signal transduction and drought resistance. However, some uncertainty remains about how CaDIMK1 regulates stress-related genes and drought response *via* ABA signaling and which downstream target proteins are phosphorylated by *CaDIMK1*. Further studies are needed to determine downstream target proteins that physically interact with and are regulated by *CaDIMK1*.

## Data Availability Statement

The original contributions presented in the study are included in the article/Supplementary Material, further inquiries can be directed to the corresponding authors.

## Author Contributions

MK, SJ, and CL performed the experiments and analyzed the results. CL and SL designed the experiments and wrote the manuscript. All authors contributed to the article and approved the submitted version.

## Conflict of Interest

The authors declare that the research was conducted in the absence of any commercial or financial relationships that could be construed as a potential conflict of interest.

## Footnotes

1 http://smart.embl-heidelberg.de

2 https://blast.ncbi.nlm.nih.gov/Blast.cgi

## Supplementary Material

Supplementary Figure 1 **(A)** Transcripts level of *CaDIMK1* in the leaves of TRV2:00 and TRV2:*CaDIMK1* plants. The *CaACT1* gene was used as an internal control for normalization. **(B)** Transcripts level of *CaDIMK1* in the *CaDIMK1-*OX *Arabidopsis*. The *Actin8* gene was used as an internal control for normalization.

Supplementary Figure 2 Increased tolerance of *CaDIMK1*-overexpressing (OX) plants to salt and osmotic stresses. **(A)** Root length of *CaDIMK1*-OX and wild-type (WT) plants on 0.5× MS medium containing 0, 400, and 500 mM Mannitol. **(B)** Root length of *CaDIMK1*-OX and wild-type plants on 0.5 MS medium containing 0, 100, 150 mM NaCl. After five days after plating, representative photographs were taken, and root lengths were measured. Values are mean ± SE with three independent experiments (*n* = 25). Asterisks indicate statistical differences between the wild-type and the transgenic plants according to the Student’s *t*-test (^∗^*P* < 0.05). The scale bar represents 1 cm.
