# Supplementary figures and images for: Mitogen-Activated Protein Kinase CaDIMK1 Functions as a Positive Regulator of Drought Stress Response and Abscisic Acid Signaling in Capsicum annuum
Source: Front Plant Sci. 2021 Apr 29;12:646707. doi: 10.3389/fpls.2021.646707 (PMC8116957; doi:10.3389/fpls.2021.646707)

A

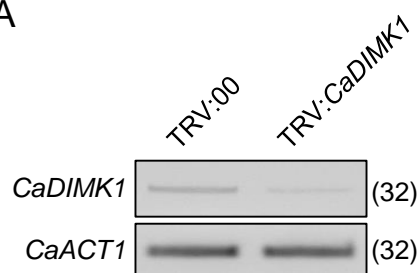

B

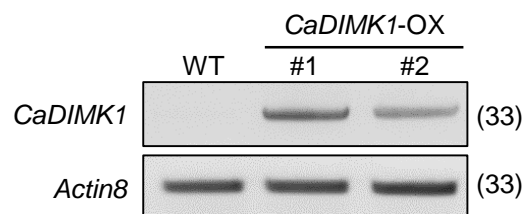

Supplementary Figure S1. Kim et al.

Supplement: Supplementary Figure 1 — (A) Transcripts level of CaDIMK1 in the leaves of TRV2:00 and TRV2:CaDIMK1 plants. The CaACT1 gene was used as an internal control for normalization. (B) Transcripts level of CaDIMK1 in the CaDIMK1-OX Arabidopsis. The Actin8 gene was used as an internal control for normalization. [file Image_1.PDF]

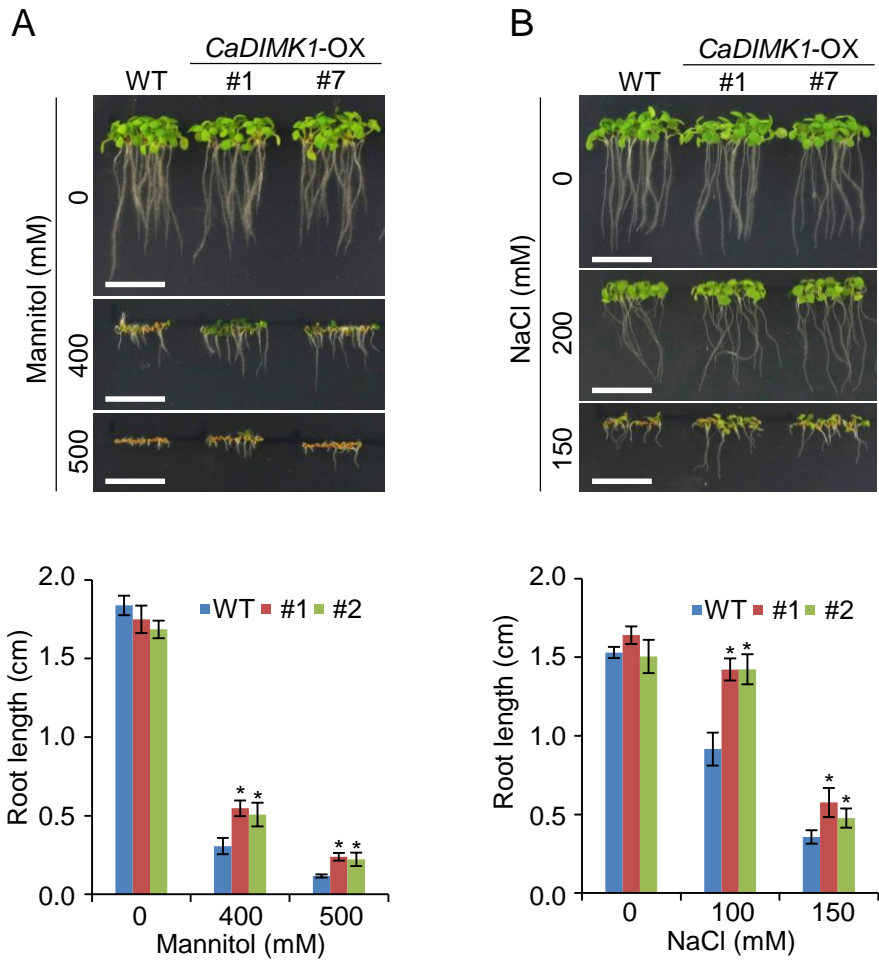

Supplement: Supplementary Figure 2 — Increased tolerance of CaDIMK1-overexpressing (OX) plants to salt and osmotic stresses. (A) Root length of CaDIMK1-OX and wild-type (WT) plants on 0.5× MS medium containing 0, 400, and 500 mM Mannitol. (B) Root length of CaDIMK1-OX and wild-type plants on 0.5 MS medium containing 0, 100, 150 mM NaCl. After five days after plating, representative photographs were taken, and root lengths were measured. Values are mean ± SE with three independent experiments (n = 25). Asterisks indicate statistical differences between the wild-type and the transgenic plants according to the Student’s t-test (∗P < 0.05). The scale bar represents 1 cm. [file Image_2.PDF]
