# Supplementary material for: Mitogen-Activated Protein Kinase CaDIMK1 Functions as a Positive Regulator of Drought Stress Response and Abscisic Acid Signaling in Capsicum annuum
Source: Front Plant Sci. 2021 Apr 29;12:646707. doi: 10.3389/fpls.2021.646707 (PMC8116957; doi:10.3389/fpls.2021.646707)
Supplement: Supplementary file 3 [file Table_1.PDF]

Supplementary Table 1. Sequences of primers used in this study.

Usage	Name		Primer sequences (5'-3')
For cloning	CaDIMK1	Forward	ATGGATTGGACTAGAGGCCA
		Reverse	TTATTCACTACTACTTCTAACTGTGAT
	VIGS	Forward	ATGGATTGGACTAGAGGCCA
		Reverse	TTATTCACTACTACTTCTAACTGTGAT
	CaDIMK1^{K32N}	Forward	CTTGGATAGCTCAACTGAGTTGACAGCAAAAACCTCATC
		Reverse	GATGAGGTTTTTGCTGTCAACTCAGTTGAGCTATCCAAG
	OST1	Forward	CCAAAGCATAGAAGAAATTATGCAG
		Reverse	TTTTGTTTGACATTTTTGTAGCAGA
For RT-PCR	CaDIMK1	Forward	ATTCAGAAACTATGGATCATTATTCAAT
		Reverse	TGTATAGTACAAAATTTACAGCAACGTG
	CaACT1	Forward	GACGTGACCTAACTGATAACCTGAT
		Reverse	CTCTCAGCA CCAATGGTAATAACTT
	RAB18	Forward	GGAAGAAGGGAATAACACAAAAGAT
		Reverse	GCGTTACAAACCCTCATTATTTTTA
	RD29B	Forward	GTTGAAGAGTCTCCACAATCACTTG
		Reverse	ATACAAATCCCCAAACTGAATAACA
	DREB2A	Forward	CTACAAAGCCTCAACTACGGAATAC
		Reverse	AAACTCGGATAGAGAATCAACAGTC
	AHG1	Forward	TCATTGATCTCAAGAATAGCTCTCA
		Reverse	TAAATAACCTTGTAGCCCATATCCA
	PP2CA	Forward	ACTTGAGGAAGAGGAGGAATAATCA
		Reverse	CCAGCCTGAATTAAGAGCTAACTAA
	HAB1	Forward	GACTACCTCTCAATGCTTGCTCTAC
		Reverse	AAAAACCTGTCGAAATTAGATCCTT
	AtActin8	Forward	CAACTATGTTCTCAGGTATTGCAGA
		Reverse	GTCATGGAAACGATGTCTCTTTAGT
